# Reconfigurable Radiation Angle Continuous Deflection of All-Dielectric Phase-Change V-Shaped Antenna

**DOI:** 10.3390/nano12193305

**Published:** 2022-09-22

**Authors:** Ping Tang, Qiao Tao, Shengde Liu, Jin Xiang, Liyun Zhong, Yuwen Qin

**Affiliations:** 1 Guangdong Provincial Key Laboratory of Information Photonics Technology, Guangdong University of Technology, Guangzhou 510006, China; 2 Guangdong Provincial Key Laboratory of Nanophotonic Functional Materials and Devices, South China Normal University, Guangzhou 510006, China; 3 School of Optoelectronic Engineering, Chongqing University, Chongqing 400044, China

**Keywords:** optical antenna, solid-state phase-change, multiple Mie mode, reconfigurable optical devices, spatial angular power splitter

## Abstract

All-dielectric optical antenna with multiple Mie modes and lower inherent ohmic loss can achieve high efficiency of light manipulation. However, the silicon-based optical antenna is not reconfigurable for specific scenarios. The refractive index of optical phase-change materials can be reconfigured under stimulus, and this singular behavior makes it a good candidate for making reconfigurable passive optical devices. Here, the optical radiation characteristics of the V-shaped phase-change antenna are investigated theoretically. The results show that with increasing crystallinity, the maximum radiation direction of the V-shaped phase-change antenna can be continuously deflected by 90°. The exact multipole decomposition analysis reveals that the modulus and interference phase difference of the main multipole moments change with the crystallinity, resulting in a continuous deflection of the maximum radiation direction. Thus, the power ratio in the two vertical radiation directions can be monotonically reversed from −12 to 7 dB between 20% and 80% crystallinity. The V-shaped phase-change antenna exhibits the potential to act as the basic structural unit to construct a reconfigurable passive spatial angular power splitter or wavelength multiplexer. The mechanism analysis of radiation directivity involving the modulus and interference phase difference of the multipole moments will provide a reference for the design and optimization of the phase-change antenna.

## 1. Introduction

The optical antenna builds the connection between the local electromagnetic field mode and the free space far-field radiation energy distribution at the sub-wavelength scale [1,2,3,4], and its applications involve advanced photon manipulation [5,6], optical communication [7,8], and biomedical sensing [9,10]. The design of a high-performance optical nanoantenna requires the simultaneous regulation of the electrical and magnetic parts of the local electromagnetic field mode to realize the high efficiency of light manipulation with controllable direction [11,12] and specific reflection or transmittance [13,14]. The local electromagnetic field mode of the nanoantenna is highly sensitive to its geometry and material composition. The direction of light can be manipulated by carefully designing specific geometric plasmonic metal nanoantennas, such as the YagI–Uda antenna [15], split-ring resonator [16], and V-shaped nanoantennas [17]. V-shaped metal nanoantennas have been used as basic structural units to construct metasurfaces and metalenses with specific properties [18,19]. In comparison to the metal nanoantennas, the all-dielectric nanoantenna with a high refractive index allows the formation of multiple Mie modes, so the radiation direction can be flexibly adjusted. Moreover, the refractive index imaginary part of the dielectric is low and the intrinsic absorption losses under the electromagnetic field are minimized, which can achieve efficient optical regulation with minimal absorption losses [13,20,21,22].

V-shaped all-dielectric silicon-based nanoantennas exhibit the efficient optical radiation characteristics of wavelength bidirectional scattering with multiple Mie modes and lower inherent ohmic loss [23]. However, as the refractive index of silicon is difficult to reconfigure in practical applications, silicon-based optical devices cannot be initialized according to specific scenarios. Unconventionally, the optical properties of all-dielectric phase-change materials can be significantly altered by solid-state phase transition [24,25,26]. The Ge-Sb-Te (GST) is a typical phase-change material, which has been exploited in a wide range of photonic devices, including optical switches [27,28], reconfigurable meta-optics [24,29,30,31,32], tunable emitters and absorbers [33,34,35,36], and nonvolatile display [37]. The nanostructure of GST can be prepared in the amorphous phase by magnetron sputtering and gradually transformed into a crystalline phase after annealing. In addition, by controlling the specific annealing temperature and time, semicrystalline states with distinct optical properties can be obtained after annealing. After the removal of the stimulus, the refractive index of GST in the amorphous, semicrystalline, and crystalline states is high and distinct, and the phase remains stable [38,39]. Recently, the optimized alloy, Ge_2_Sb_2_Se_4_Te_1_ (GSS4T1), combines broadband transparency (1–18.5 μm), large optical contrast (Δn = 2.0), and significantly improved glass forming ability, making it a better candidate for reconfigurable passive optical devices [40]. The flexibility, compatibility, and passivity of optical devices based on all-dielectric phase-change materials make them very suitable for optical applications [41,42].

In this paper, the feasibility of the V-shaped GSS4T1 antenna for reconfigurable radiation angular power splitter is explored, and variation of the optical radiation angle with phase-change crystallinity is theoretically investigated. For micro/nanostructures of phase-change materials, strong absorption based on anapole mode and full backward or forward scattering based on the Kerker condition has been studied [28,36]. Herein, we systematically analyze the radiation angle continuous deflection of the phase-change antenna, including the influence of each scattering multipole moment with different modulus and phase angle. By the finite element method (FEM) and current density-based multipole decomposition [43,44], the relationship between the continuous deflection of the antenna radiation directivity and the change of multipole moments with the crystallinity is investigated. The results show that the maximum radiation direction of the V-shaped phase-change antenna can continuously be deflected by about 90° with the material phase change. The power ratio in two vertical radiation directions can be monotonically reversed from −12 to 7 dB between 20% and 80% crystallinity. Multipole decomposition reveals that the continuous deflection of radiation direction of V-shaped phase-change antenna with crystallinity is due to the change of complex coefficient of the main multipole moment, including modulus and interference phase difference. Especially, the interference phase differences of main multipole moments are the key to the radiation direction continuous deflection. Finally, the consistency of the far-field radiation pattern reconstructed from the multipole scattering coefficient and the one calculated by FEM demonstrates the reliability of the mechanism analysis. We designed the V-shaped phase-change antenna as a promising candidate for reconfigurable passive spatial angular power splitter or wavelength multiplexer.

## 2. Theoretical and Methods

**Figure 1:**
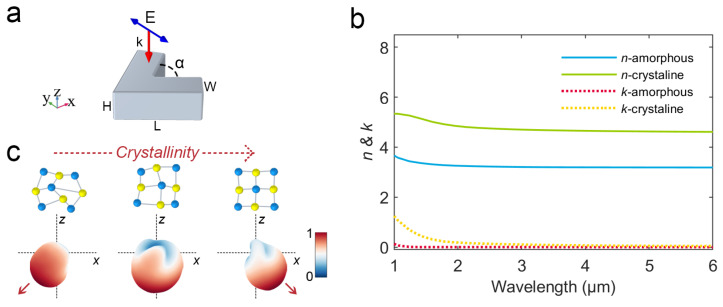
Calculation model and refractive index parameters of phase-change material: (**a**) Schematic diagram of the V-shaped phase-change antenna geometry and the incident light direction; (**b**) Complex refractive index of amorphous (0%) and crystalline (100%) GSS4T1 phase-change materials; (**c**) Conceptual illustration of V-shaped phase-change antenna for continuous reconfigurable radiation deflection.

To investigate the feasibility of a V-shaped phase-change antenna for a reconfigurable radiation angular power splitter, the numerical calculation of the electromagnetic field is performed based on the FEM with commercially available software (COMSOL Multiphysics 5.6, COMSOL Inc., Sweden). As shown in Figure 1a, the V-shaped phase-change antenna is symmetric about the *x*-axis with its center section in the *xy*-plane, in which the length *L* is 2.0 μm, the width *W* is 0.70 μm, the height *H* is 0.75 μm, and the included angle α is 75°. A *y*-polarized plane light wave with amplitude $E_{0}$ = 1 V/m propagates along the −z-direction. The antenna is embedded in a homogeneous air host medium with relative permittivity $\varepsilon_{\mathrm{air}}$ = 1. Taking the perfect matching layer (PML) as the boundary condition, the Helmholtz equation of electric field E is calculated [45]:

(1) $$\nabla \times \left(\nabla \times E\right)-k_{0}^{2}\varepsilon_{r}E=0,$$

where $k_{0}$ is the wave vector and $\varepsilon_{r}={\left(n-ik\right)}^{2}$. The *n* and *k* are the real and imaginary parts of the complex refractive index of the antenna material, respectively. As shown in Figure 1b, the complex refractive index of amorphous and crystalline GSS4T1 phase-change materials is the fitting value of the experimental data of GSS4T1 in Ref. [40]. In addition, the permittivity of GSS4T1 varies with crystallinity *C* using the following relation [36]:

(2) $$\frac{\varepsilon_{\mathrm{GSS}4T1}\left(\lambda ,C\right)-1}{\varepsilon_{\mathrm{GSS}4T1}\left(\lambda ,C\right)+2}=C\times \frac{\varepsilon_{\mathrm{cGSS}4T1}\left(\lambda \right)-1}{\varepsilon_{\mathrm{cGSS}4T1}\left(\lambda \right)+2}+\left(1-C\right)\times \frac{\varepsilon_{\mathrm{aGSS}4T1}\left(\lambda \right)-1}{\varepsilon_{\mathrm{aGSS}4T1}\left(\lambda \right)+2},$$

where $\varepsilon_{\mathrm{aGSS}4T1}$ and $\varepsilon_{\mathrm{cGSS}4T1}$ are the permittivities of amorphous (0%) and crystalline (100%) GSS4T1, respectively. Figure 1c shows the concept that, for the V-shaped phase-change antenna with a fixed geometric size, its radiation directivity can be continuously reconfigured by adjusting the crystallinity with stimulus.

The numerically calculated scattering field is the difference between the total field and the incident light field:

(3) $$E_{\mathrm{scat}}=E-E_{\mathrm{inc}},$$

(4) $$H_{\mathrm{scat}}=H-H_{\mathrm{inc}}.$$

According to the above scattering field, the scattering cross section can be calculated by using the following relations [46]:

(5) $$P_{\mathrm{scat}}=\int n\cdot P_{\mathrm{scat}}dS,$$

(6) $$P_{\mathrm{inc}}=\frac{1}{2\eta }{\left|E_{\mathrm{inc}}\right|}^{2},$$

(7) $$\sigma_{\mathrm{scat}\;}=\frac{P_{\mathrm{scat}}}{P_{\mathrm{inc}}},$$

where $P_{\mathrm{scat}}$ is the Poynting vector of the scattered field, n is the unit normal vector of the far-field boundary S, and $\eta =\sqrt{\mu_{0}}/\varepsilon_{0}$.

Based on the scattering field, the Stratton–Chu formula is adopted to calculate the far-field radiation electric field of the angular point *p* [47]:

(8) $$E_{\mathrm{far}\to p}=\frac{ik_{0}}{4\pi }n_{r}\times \int \left[n\times E_{\mathrm{scat}}-\eta n_{r}\times \left(n\times H_{\mathrm{scat}}\right)\right]e^{ik_{0}r\cdot n_{r}}dS,$$

where $n_{r}$ is the unit vector in the direction of the radius vector r.

According to the far-field intensity I(θ,φ), the directivity of the positive and negative *x*-axis is calculated by:

(9) $$X/-X=10\log_{10}\frac{{\;\int \int \;}_{\left(0,-\frac{\pi }{2}\right)}^{\left(\pi ,\frac{\pi }{2}\right)}I\left(\theta ,\varphi \right)\sin\left(\theta \right)d\varphi d\theta }{{\;\int \int \;}_{\left(0,\frac{\pi }{2}\right)}^{\left(\pi ,\frac{3\pi }{2}\right)}I\left(\theta ,\varphi \right)\sin\left(\theta \right)d\varphi d\theta },$$

the directivity of the positive and negative *z*-axis is calculated by:

(10) $$Z/-Z=10\log_{10}\frac{{\;\int \int \;}_{\left(0,0\right)}^{\left(\frac{\pi }{2},2\pi \right)}I\left(\theta ,\varphi \right)\sin\left(\theta \right)d\varphi d\theta }{{\;\int \int \;}_{\left(\frac{\pi }{2},0\right)}^{\left(\pi ,2\pi \right)}I\left(\theta ,\varphi \right)\sin\left(\theta \right)d\varphi d\theta },$$

and the directivity of specific radiation angle and window size is calculated by:

(11) $$D=10\log_{10}\frac{{\;\int \int \;}_{\left(\theta_{0}-\delta ,\pi -\delta \right)}^{\left(\theta_{0}+\delta ,\pi +\delta \right)}I\left(\theta ,\varphi \right)\sin\left(\theta \right)d\varphi d\theta }{{\;\int \int \;}_{\left(\theta_{0}-\delta ,-\delta \right)}^{\left(\theta_{0}+\delta ,+\delta \right)}I\left(\theta ,\varphi \right)\sin\left(\theta \right)d\varphi d\theta },$$

where $\theta_{0}$ and δ are taken to be 135° and 10°, respectively.

It is difficult to clarify the physical mechanism by the numerically calculated results, and the scattering multipole decomposition is an essential theoretical analysis for the in-depth study of the radiation mechanism of antennas. Beyond the long-wavelength approximation, the exact expressions for the multipole moments are valid for any wavelength and size dimensions [43,44]. To clarify the mechanism of the variation of the radiation angle of the V-shaped phase-change antenna with the crystallinity, the multipole decomposition with exact expressions is performed. Firstly, the current density can be calculated according to E:

(12) $$J\left(r\right)=-i\omega \varepsilon_{0}\left[\varepsilon_{r}\left(r\right)-\varepsilon_{\mathrm{air}}\right]E\left(r\right).$$

Here, the dipole and quadrupole are mainly considered. Then, we calculate the electric dipole (ED), magnetic dipole (MD), electric quadrupole (EQ), and magnetic quadrupole (MQ) by the exact expressions:

(13) $$p_{\alpha }=\frac{i}{\omega }\left\{\int rJ_{\alpha }j_{0}\left(k_{0}r\right)dv+\frac{k_{0}^{2}}{2}\int \left[3\left(r\cdot J\right)r_{\alpha }-r^{2}J_{\alpha }\right]\frac{j_{2}\left(k_{0}r\right)}{{\left(k_{0}r\right)}^{2}}dv\right\},$$

(14) $$m_{\alpha }=\frac{3}{2}\int {\left(r\times J\right)}_{\alpha }\frac{j_{1}\left(k_{0}r\right)}{k_{0}r}dv,$$

(15) $$\begin{array}{cc} Q_{\alpha \beta }^{e} & =\frac{3i}{\omega }\left\{\int \left[3\left(r_{\beta }J_{\alpha }+r_{\alpha }J_{\beta }\right)-2\left(r\cdot J\right)\delta_{\alpha \beta }\right]\frac{j_{1}\left(k_{0}r\right)}{k_{0}r}dv\right.\\ \; & \left.+2k_{0}^{2}\int \left[5r_{\alpha }r_{\beta }\left(r\cdot J\right)-\left(r_{\alpha }J_{\beta }+r_{\beta }J_{\alpha }\right)r^{2}-r^{2}\left(r\cdot J\right)\delta_{\alpha \beta }\right]\frac{j_{3}\left(k_{0}r\right)}{{\left(k_{0}r\right)}^{3}}dv\right\} \end{array},$$

(16) $$Q_{\alpha \beta }^{m}=15\int \left\{r_{\alpha }{\left(r\times J\right)}_{\beta }+r_{\beta }{\left(r\times J\right)}_{\alpha }\right\}\frac{j_{2}\left(k_{0}r\right)}{{\left(k_{0}r\right)}^{2}}dv,$$

where α,β=x,y,z, and $j_{n}\left(\rho \right)$ denotes the spherical Bessel function. Using the multipole moments, the sum of the scattering contributions from different multipole moments is written as [43]:

(17) $$\begin{array}{cc} C_{\mathrm{scat}}^{\mathrm{sum}\;} & =C_{\mathrm{scat}}^{p}+C_{\mathrm{scat}}^{m}+C_{\mathrm{scat}}^{Q^{e}}+C_{\mathrm{scat}}^{Q^{m}}+\cdots \\ \; & =\frac{k_{0}^{4}}{6\pi \varepsilon_{0}^{2}{\left|E_{\mathrm{inc}}\right|}^{2}}\left[\sum_{\alpha }\left({\left|p_{\alpha }\right|}^{2}+{\left|\frac{m_{\alpha }}{c_{0}}\right|}^{2}\right)\right.\\ + & \left.\frac{1}{120}\sum_{\alpha \beta }\left({\left|k_{0}Q_{\alpha \beta }^{e}\right|}^{2}+{\left|\frac{k_{0}Q_{\alpha \beta }^{m}}{c_{0}}\right|}^{2}\right)+\cdots \right] \end{array}.$$

The scattering far-field from the V-shaped phase-change antenna described up to quadrupole order in Cartesian coordinates can be defined as [44]:

(18) $$\begin{array}{cc} E_{\mathrm{far}}= & \frac{k_{0}^{2}}{4\pi \varepsilon_{0}}e^{ik_{0}R}\frac{1}{R}\left(\alpha_{{\mathrm{ED}}_{\alpha }}\left[n_{r}\times \left[n_{\alpha }\times n_{r}\right]\right]\right.\\ \; & +\alpha_{{\mathrm{MD}}_{\alpha }}\left[n_{\alpha }\times n_{r}\right]\\ + & \alpha_{{\mathrm{EQ}}_{\alpha \beta }}\left[n_{r}\times \left[n_{r}\times n_{\alpha }n_{\beta }\cdot n_{r}\right]\right]\\ \; & \left.+\alpha_{{\mathrm{EQ}}_{\alpha \beta }}\left[n_{r}\times n_{\alpha }n_{\beta }\cdot n_{r}\right]\right), \end{array}$$

(19) $$\alpha_{{\mathrm{ED}}_{\alpha }}=p_{\alpha },$$

(20) $$\alpha_{{\mathrm{MD}}_{\alpha }}=-\frac{1}{c_{0}}m_{\alpha },$$

(21) $$\alpha_{{\mathrm{EQ}}_{\alpha \beta }}=-\frac{ik_{0}}{6}Q_{\alpha \beta }^{e},$$

(22) $$\alpha_{{\mathrm{MQ}}_{\alpha \beta }}=\frac{ik_{0}}{6c_{0}}Q_{\alpha \beta }^{m},$$

where *R* = 1 m is the radius of the far-field radiation receiving spherical surface, $\alpha_{{\mathrm{ED}}_{\alpha }}$, $\alpha_{{\mathrm{MD}}_{\alpha }}$, $\alpha_{{\mathrm{EQ}}_{\alpha \beta }}$, and $\alpha_{{\mathrm{EQ}}_{\alpha \beta }}$ are the complex coefficients of the multipole moments.

## 3. Results and Discussion

**Figure 2:**
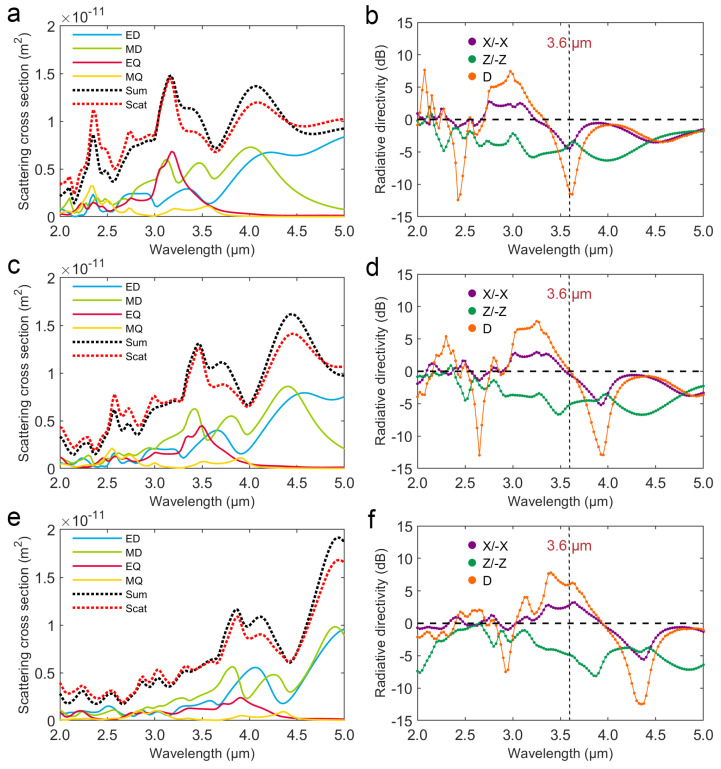
Multipole scattering cross sections and directivities of the V-shaped phase-change antenna in the 2.0 to 5.0 μm wavelength range: (**a**,**c**,**e**) Scattering cross sections of ED, MD, EQ, MQ, their summation (Sum), and the total scattering cross sections calculated according to scattering field (Scat) at the crystallinity of 20%, 50% and 80%, respectively; (**b**,**d**,**f**) Directivities of *x*-axis positive and negative (X/−X), *z*-axis positive and negative (Z/−Z), and the specific angle and window (D: $\theta_{0}$ = 135°, δ = 10°) at the crystallinity of 20%, 50% and 80%, respectively.

Firstly, we calculate the electromagnetic field of a V-shaped phase-change antenna in the wavelength range of 2.0 to 5.0 μm at the crystallinity of 20%, 50% and 80%. Then, multipole decomposition based on the current density is performed to analyze the antenna radiation. The scattering cross sections of ED, MD, EQ, MQ, their summations (Sum), and the total scattering cross sections calculated from the scattering field (Scat) at the crystallinity of 20%, 50%, and 80% in the wavelength range of 2.0 to 5.0 μm are shown in Figure 2a,c,e, respectively. It can be seen that the peak shapes of Sum and Scat are almost the same, which indicates that the multipole decomposition described up to the quadrupole order is reliable. The multipole scattering cross sections are redshifted with increasing crystallinity, which results from the refractive index of GSS4T1 increasing with crystallinity. The electric and magnetic field distributions in the *xy*-plane of the V-shaped phase-change antenna are shown in Figure S1. It shows that at the 3.6 μm wavelength, the V-shaped antennas with crystallinity of 20%, 50% and 80% produce different near-field electromagnetic resonance modes. It leads to different far-field scattering. Consequently, based on the calculated scattering field, the directivities of V-shaped antennas at the crystallinity of 20%, 50% and 80% are calculated, including the *x*-axis positive-negative (X/−X) directivity, *z*-axis positive-negative (Z/−Z) directivity, as well as the specific angle and window size (D: $\theta_{0}$ = 135°, δ = 10°) directivity, which are shown in Figure 2b,d,f, respectively. Obviously, the three directivity curves are redshifted with increasing crystallinity. Note that for V-shaped phase-change antennas at 3.6 μm wavelength, when the crystallinity increases between 20% and 80%, the X/−X or D directivity reverses, while the Z/−Z directivity is almost negative. In particular, the D directivity could be reversed from −12 dB to 7 dB by changing the crystallinity at 3.6 μm wavelength. Furthermore, multipole scattering cross sections and directivities of the amorphous and crystalline V-shaped phase-change antennas are shown in Figure S2. For the amorphous (0%) V-shaped antenna, the X/−X or D directivity reverses in the wavelength range of 2.8 to 3.4 μm (Figure S2b). In addition, for crystalline (100%) V-shaped antennas, the X/−X or D directivity reverses approximately in the wavelength range of 4.0 to 4.7 μm (Figure S2d). These results suggest that X/−X or D directivity could be reversed by changing the crystallinity at a selected specific wavelength in the intersecting range of 3.4 to 4.0 μm.

**Figure 3:**
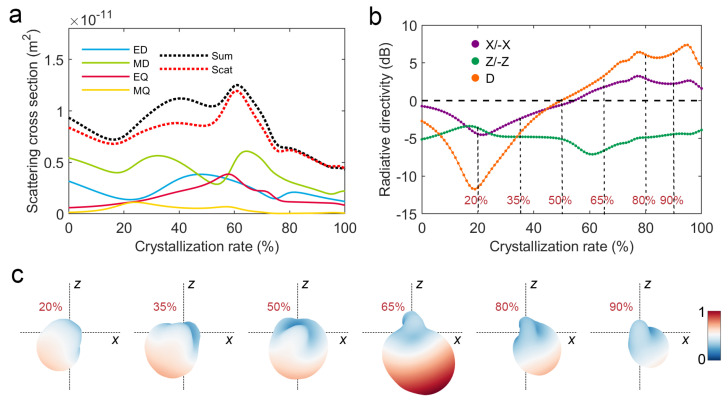
Scattering characteristics of V-shaped phase-change antenna at 3.6 μm wavelength: (**a**) Continuous changes of multipole scattering cross sections with the crystallinity; (**b**) Continuous changes of X/−X directivity, Z/−Z directivity, and D directivity with the crystallinity; (**c**) Modulus normalized far-field radiation patterns in crystallinity of 20%, 35%, 50%, 65%, 80% and 90%, respectively.

To further investigate the continuous change of the V-shaped phase-change antenna’s scattering with the crystallinity, we calculated the multipole scattering cross sections and directivities of the V-shaped phase-change antenna in the crystallinity between 0% and 100% at 3.6 μm wavelength, which are shown in Figure 3a,b, respectively. It can be seen that the X/−X directivity reverses monotonically from −12 to 7dB in the range of 20% to 80% crystallinity. Based on the scattering field, the Stratton–Chu formula is used to calculate the far-field radiation of the V-shaped phase-change antenna at the crystallinity of 20%, 35%, 50%, 65%, 80% and 90%, and the modulus normalized results are shown in Figure 3c. Obviously, the maximum radiation direction of the V-shaped phase-change antenna reverses about 90° with an increase in crystallinity. The above results fully reflect the theoretical feasibility of realizing continuous controllable angular power splitting of a V-shaped phase-change antenna based on reconfigurable phase transition.

**Figure 4:**
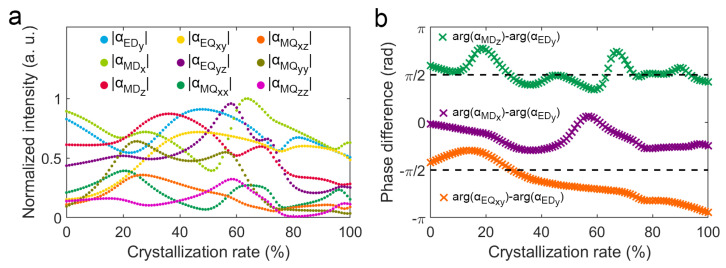
Multipole moments at 3.6 μm wavelength: (**a**) Normalized moduli of multipole moments as functions of the crystallinity; (**b**) Interference phase differences of multipole moments as functions of the crystallinity.

To clarify the mechanism of radiation directivity change of the V-shaped phase-change antenna, we deeply analyze the change of multipole moments with crystallinity. The calculation results indicate that non-zero multipole moments include ${\mathrm{ED}}_{y}$, ${\mathrm{MD}}_{x}$, ${\mathrm{MD}}_{z}$, ${\mathrm{EQ}}_{\mathrm{xy}}$, ${\mathrm{EQ}}_{\mathrm{yz}}$, ${\mathrm{MQ}}_{\mathrm{xx}}$, ${\mathrm{MQ}}_{\mathrm{xz}}$, ${\mathrm{MQ}}_{\mathrm{yy}}$, and ${\mathrm{MQ}}_{\mathrm{zz}}$. The complex coefficient of each multipole moment includes the modulus and phase angle. The modulus determines the radiation amplitude of the multipole moment, the normalized modulus of complex coefficients of these multipole moments are shown in Figure 4a. It can be seen that $\alpha_{{\mathrm{ED}}_{y}}$, $\alpha_{{\mathrm{MD}}_{x}}$, $\alpha_{{\mathrm{MD}}_{z}}$, $\alpha_{{\mathrm{EQ}}_{xy}}$, and $\alpha_{{\mathrm{EQ}}_{yz}}$ are relatively large, while $\alpha_{{\mathrm{EQ}}_{xx}}$, $\alpha_{{\mathrm{EQ}}_{xz}}$, and $\alpha_{{\mathrm{EQ}}_{zz}}$ are relatively small, indicating that ${\mathrm{ED}}_{y}$, ${\mathrm{MD}}_{x}$, ${\mathrm{MD}}_{z}$, ${\mathrm{EQ}}_{\mathrm{xy}}$, and ${\mathrm{EQ}}_{\mathrm{yz}}$ make relatively large contributions to the far-field radiation of the V-shaped phase change antenna, while ${\mathrm{MQ}}_{\mathrm{xx}}$, ${\mathrm{MQ}}_{\mathrm{xz}}$, and ${\mathrm{MQ}}_{\mathrm{zz}}$ make relatively small contributions. In addition, the intrinsic far-field radiation patterns of the unit multipole moments can be seen in Figure S3. D directivity is the key of a V-shaped phase-change antenna to achieve a continuous reconfigurable radiation angular power continuous control, which is closely related to the ratio of the radiation modulus in the two directions of (θ: 135°, φ: 0°) and (θ: 135°, φ: 180°) which can be simplified from Equation (18):

(23) $$\frac{\left|E_{\mathrm{far}}\left(\theta :135^{\circ },\varphi :0^{\circ }\right)\right|}{\left|E_{\mathrm{far}}\left(\theta :135^{\circ },\varphi :180^{\circ }\right)\right|}=\frac{\left|\alpha_{{\mathrm{ED}}_{y}}+\frac{\alpha_{{\mathrm{MD}}_{z}}}{\sqrt{2}}-\frac{\alpha_{{\mathrm{EQ}}_{xy}}}{\sqrt{2}}+\frac{\alpha_{{\mathrm{EQ}}_{yz}}}{\sqrt{2}}-\frac{\alpha_{{\mathrm{EQ}}_{xx}}}{2}+\frac{\alpha_{{\mathrm{EQ}}_{zz}}}{2}\right|}{\left|\alpha_{{\mathrm{ED}}_{y}}-\frac{\alpha_{{\mathrm{MD}}_{z}}}{\sqrt{2}}+\frac{\alpha_{{\mathrm{EQ}}_{xy}}}{\sqrt{2}}+\frac{\alpha_{{\mathrm{EQ}}_{yz}}}{\sqrt{2}}+\frac{\alpha_{{\mathrm{EQ}}_{xx}}}{2}-\frac{\alpha_{{\mathrm{EQ}}_{zz}}}{2}\right|}.$$

This formula suggests the multipole moments that affect D directivity are ${\mathrm{ED}}_{y}$, ${\mathrm{MD}}_{z}$, ${\mathrm{EQ}}_{\mathrm{xy}}$, ${\mathrm{EQ}}_{\mathrm{yz}}$, ${\mathrm{MQ}}_{\mathrm{xx}}$, and ${\mathrm{MQ}}_{\mathrm{zz}}$. Comparing the far-field modulus in the upper and lower of above fractions, the coefficients of $\alpha_{{\mathrm{ED}}_{y}}$ and $\alpha_{{\mathrm{EQ}}_{yz}}$ are the same, while the coefficient of $\alpha_{{\mathrm{MD}}_{z}}$, $\alpha_{{\mathrm{EQ}}_{xy}}$, $\alpha_{{\mathrm{EQ}}_{xx}}$, and $\alpha_{{\mathrm{EQ}}_{zz}}$ are opposite. This indicates that ${\mathrm{MD}}_{z}$, ${\mathrm{EQ}}_{\mathrm{xy}}$, ${\mathrm{MQ}}_{\mathrm{xx}}$, and ${\mathrm{MQ}}_{\mathrm{zz}}$ lead to the radiation difference in the above two directions and are the key moments in the direction change of lateral deflection. The multipole moments interference forms the final far-field radiation pattern, and the modulus and interference phase difference of the multipole scattering coefficient together determine the final far-field radiation pattern.

The phase angle differences between the interference multipole moments are critical to the direction of far-field radiation. To investigate how the interference phase difference of multipole moments affect far-field radiation patterns, we calculate the interference far-field radiation patterns of the unit multipole moments with different phase differences (see Figure S4). In the Supplementary Material, we have deeply analyzed and compared the influence of each multipole moment on the far-field radiation pattern, and the related analysis clearly indicates that ${\mathrm{ED}}_{y}$, ${\mathrm{MD}}_{x}$, ${\mathrm{MD}}_{z}$, and ${\mathrm{EQ}}_{\mathrm{xy}}$ make the major contributions to the change of the D directivity of the V-shaped antenna. Consequently, the interference phase differences between ${\mathrm{MD}}_{x}$ and ${\mathrm{ED}}_{y}$, ${\mathrm{MD}}_{z}$ and ${\mathrm{ED}}_{y}$, and ${\mathrm{EQ}}_{\mathrm{xy}}$ and ${\mathrm{ED}}_{y}$ with crystallinity from 0% to 100% have been calculated and shown in Figure 4b. It shows that interference phase differences between ${\mathrm{MD}}_{x}$ and ${\mathrm{ED}}_{y}$, ${\mathrm{MD}}_{z}$ and ${\mathrm{ED}}_{y}$, and ${\mathrm{EQ}}_{\mathrm{xy}}$ and ${\mathrm{ED}}_{y}$ vary differently with crystallinity. In addition, the interference phase difference of ${\mathrm{MD}}_{x}$ and ${\mathrm{ED}}_{y}$ mainly change in the range of −π/4 to π/4, which produces forward scattering along the *z*-axis as shown in Figure S4.

**Figure 5:**
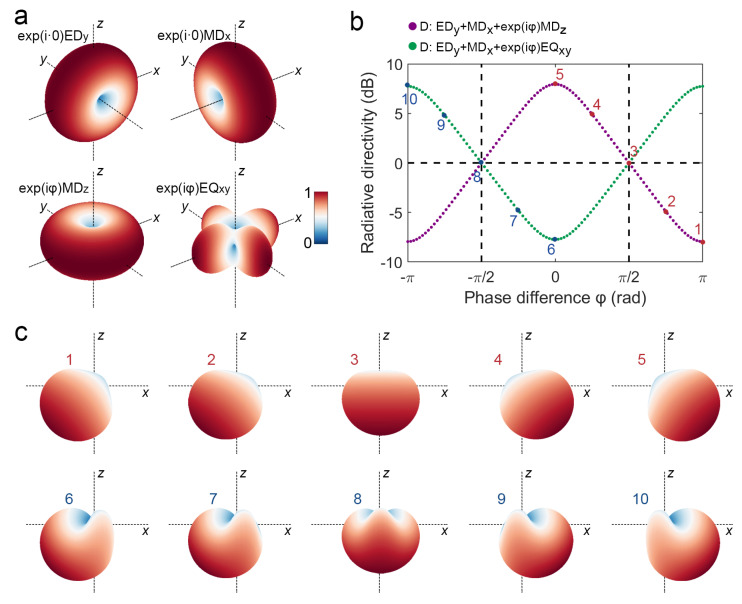
Directional deflection of far-field radiation with an interference phase difference: (**a**) Far-field radiation pattern of unit ${\mathrm{ED}}_{y}$ and ${\mathrm{MD}}_{x}$ with phase angle 0, ${\mathrm{MD}}_{z}$ and ${\mathrm{EQ}}_{\mathrm{xy}}$ with phase angle φ; (**b**) D directivity varies with the interference phase difference φ; (**c**) Far-field radiation patterns corresponding the points numbered 1–10 in (**b**), respectively.

To understand how interference phase differences cause the radiation angle continuous deflection of V-shaped phase-change antenna, we first analyze the interference far-field radiation of unit ${\mathrm{ED}}_{y}$ and ${\mathrm{MD}}_{x}$ with phase angle 0 (i.e., $\alpha_{{\mathrm{ED}}_{y}},\alpha_{{\mathrm{MD}}_{x}}=\exp\left(i\cdot 0\right)$), unit ${\mathrm{MD}}_{z}$ and ${\mathrm{EQ}}_{\mathrm{xy}}$ with phase angle φ (i.e., $\alpha_{{\mathrm{ED}}_{y}},\alpha_{{\mathrm{MD}}_{x}}=\exp\left(i\varphi \right)$). As shown in Figure 5a, the intrinsic far-field radiation patterns of unit ${\mathrm{ED}}_{y}$, ${\mathrm{MD}}_{x}$, ${\mathrm{MD}}_{z}$, and ${\mathrm{EQ}}_{\mathrm{xy}}$ do not vary with their respective phase angle, but when they interfere with each other, their phase difference causes changes in the direction of the final far-field radiation. As shown in Figure 5b, it can be seen that both D directivities of ${\mathrm{ED}}_{y}$+${\mathrm{MD}}_{x}$+$\exp\left(i\varphi \right){\mathrm{MD}}_{z}$ and ${\mathrm{ED}}_{y}$+${\mathrm{MD}}_{x}$+$\exp\left(i\varphi \right){\mathrm{EQ}}_{\mathrm{xy}}$ reverse at the phase difference of −π/2 and π/2. In addition, the interference far-field radiation patterns corresponding to the points numbered 1–10 in Figure 4b are shown in Figure 5c. As shown in Figure 4b, the calculated phase differences of ${\mathrm{MD}}_{z}$ and ${\mathrm{ED}}_{y}$ continuously change around φ=π/2 with crystallinity; when the crystallinity is at 20%, the phase difference is about 3π/4 and the far-field radiation contribution of ${\mathrm{MD}}_{z}$ corresponds to case number 2 in Figure 5c; and when crystallinity is at 50%, the phase difference is about π/2 and the far-field radiation contribution of ${\mathrm{MD}}_{z}$ corresponds to case number 3 in Figure 5c. In contrast, the calculated phase differences of ${\mathrm{EQ}}_{\mathrm{xy}}$ and ${\mathrm{ED}}_{y}$ continuous changes around φ=−π/2 with crystallinity; and when the crystallinity increases in the range of 20% to 80%, the far-field radiation contribution of ${\mathrm{EQ}}_{\mathrm{xy}}$ corresponds to the cases number 7 to 10 in Figure 5c. Furthermore, ${\mathrm{MD}}_{z}$ and ${\mathrm{EQ}}_{\mathrm{xy}}$ contribute in the same direction near 20% crystallinity, while ${\mathrm{MD}}_{z}$ and ${\mathrm{EQ}}_{\mathrm{xy}}$ contribute in the opposite direction near 60% crystallinity. That explains why the D directivity calculated by FEM has a significant change trend of about 20% crystallinity, but it changes slowly at about 80% crystallinity (see Figure 3b).

**Figure 6:**
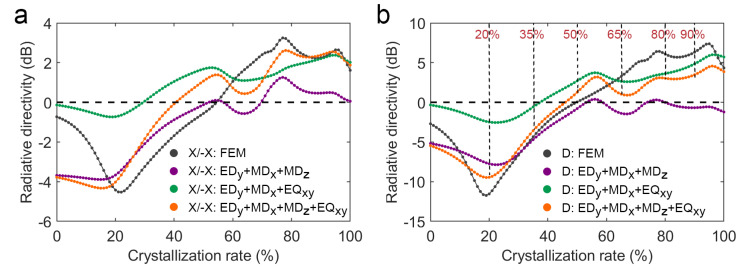
Directivities of the FEM results and interference far-field radiation patterns according to the complex coefficients of major multipole moments at 3.6 μm wavelength: (**a**) X/−X directivity as a function of crystallinity; (**b**) D directivity as a function of crystallinity.

According to the calculated complex coefficients of the major multipole moments, the X/−X and D directivities of the interference far-field radiation have been obtained, which are shown in Figure 6. It is found that the interference of ${\mathrm{ED}}_{y}$, ${\mathrm{MD}}_{x}$, and ${\mathrm{MD}}_{z}$ are in good agreement with the calculated results in the crystallinity range of 0% to 50%, while the crystallinity range of 50% to 100% is quite different from the FEM result. In contrast, the interference of ${\mathrm{ED}}_{y}$, ${\mathrm{MD}}_{x}$, and ${\mathrm{EQ}}_{\mathrm{xy}}$ in the crystallinity range of 50% to 100% is relatively consistent with the calculated result, while the crystallinity range of 0% to 50% is quite different from the FEM result. Moreover, the interference of ${\mathrm{ED}}_{y}$, ${\mathrm{MD}}_{x}$, ${\mathrm{MD}}_{z}$, and ${\mathrm{EQ}}_{\mathrm{xy}}$ is more consistent with the FEM result in the whole crystallinity range. These results indicate that the V-shaped antenna’s D directivity that changes continuously from 0% to 50% crystallinity is mainly the contribution of ${\mathrm{MD}}_{z}$, and its D directivity that changes continuously from 50% to 100% crystallinity is mainly the contribution of ${\mathrm{EQ}}_{\mathrm{xy}}$. The above analysis shows that the continuous change in radiation direction of V-shaped phase-change antenna with crystallinity is due to the change of complex coefficient of the main multipole moment ${\mathrm{ED}}_{y}$, ${\mathrm{MD}}_{x}$, ${\mathrm{MD}}_{z}$, and ${\mathrm{EQ}}_{\mathrm{xy}}$, including modulus and interference phase difference. Moreover, we also consider the influence of the minor moments on the directivities of interference far-field radiation (see Figure S5). Although the minor moments ${\mathrm{MQ}}_{\mathrm{xx}}$ and ${\mathrm{MQ}}_{\mathrm{zz}}$ cannot cause significant changes in directivities, it shows a tendency to approach the FEM results.

**Figure 7:**
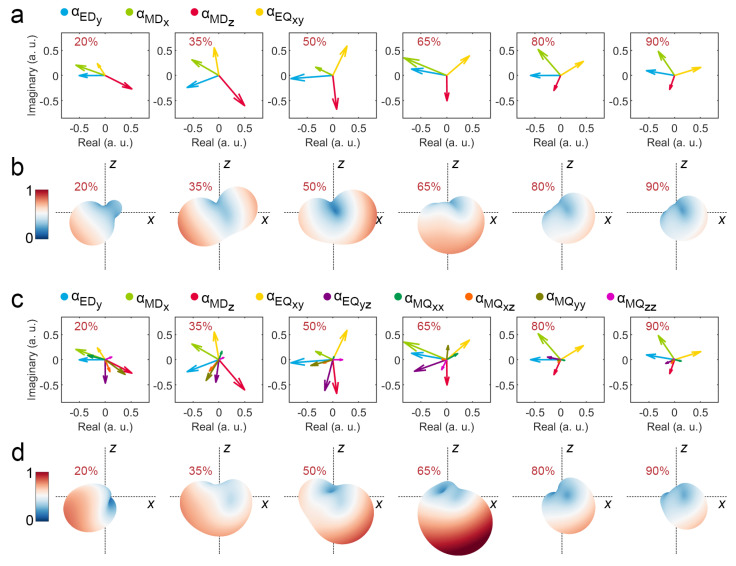
Interference far-field radiation patterns reconstructed by calculated complex multipole scattering coefficients in crystallinities of 20%, 35%, 50%, 65%, 80% and 90%: (**a**) Modulus normalized complex multipole coefficients and (**b**) corresponding reconstructed far-field radiation of moments with major contribution to D directivity; (**c**) Modulus normalized complex multipole coefficients and (**d**) corresponding reconstructed far-field radiation of all non-zero moments.

To verify the reliability of the above multipole scattering analysis, we use the calculated multipole scattering coefficients to reconstruct the interference far-field radiation pattern and compare it with the far-field radiation pattern calculated by FEM. For the V-shaped phase-change antenna at a wavelength of 3.6 μm with crystallinity of 20%, 35%, 50%, 65%, 80% and 90%, the multipole moments ${\mathrm{ED}}_{y}$, ${\mathrm{MD}}_{x}$, ${\mathrm{MD}}_{z}$, and ${\mathrm{EQ}}_{\mathrm{xy}}$ make a major contribution to D directivity, and their modulus normalized coefficients are expressed in the complex coordinate system (Figure 7a), and the corresponding reconstructed interference far-field radiation patterns are shown in Figure 7b. Obviously, the relative change of radiation in (θ: 135°, φ: 0°) and (θ: 135°, φ: 180°) shows the angular power splitting function of the V-shaped phase-change antenna, which can be reconfigured by the controllable phase transition. However, because multipole moments in other directions are not considered, the reconstructed far-field radiation pattern is different from the far-field radiation pattern calculated by FEM (Figure 3c). Correspondingly, we further consider all the above non-zero multipole moments and express their modulus normalized scattering coefficients in the complex coordinate system (Figure 7c), and the corresponding reconstructed interference far-field radiation patterns are shown in Figure 7d. Obviously, the reconstructed far-field radiation pattern considering all multipole moments is close to the result of the FEM calculation (Figure 3c). This comparison fully demonstrates the reliability of the above mechanism analysis of multipole scattering.

In addition, D directivities of the V-shaped phase change antenna with different geometric angles (α in Figure 1a), including α = 60°, α = 65°, α = 70°, α = 75°, and α = 80° have been calculated and shown in Figure S6. It reveals that by reducing the geometric angle, a greater power ratio in the direction (θ: 135°, φ: 180°) can be achieved.

## 4. Conclusions

The radiation direction of the V-shaped phase-change antenna deflects continuously by 90° with increasing crystallinity. In-depth analysis of multipole decomposition reveals that ${\mathrm{ED}}_{y}$, ${\mathrm{MD}}_{x}$, ${\mathrm{MD}}_{z}$, and ${\mathrm{EQ}}_{\mathrm{xy}}$ make the major contributions to the change in D directivity of the V-shaped antenna. In addition, the continuous change in radiation direction of V-shaped phase-change antenna with crystallinity is due to the change in the complex coefficient of the main multipole moment ${\mathrm{ED}}_{y}$, ${\mathrm{MD}}_{x}$, ${\mathrm{MD}}_{z}$, and ${\mathrm{EQ}}_{\mathrm{xy}}$, including the modulus and interference phase difference. In particular, the interference phase differences between ${\mathrm{MD}}_{z}$ and ${\mathrm{ED}}_{y}$, and between ${\mathrm{EQ}}_{\mathrm{xy}}$ and ${\mathrm{ED}}_{y}$ that change with crystallinity cause the radiation angle continuous deflection of V-shaped phase-change antenna. The D directivity of the V-shaped phase-change antenna can be monotonically reversed from −12 to 7 dB in a crystallinity of 20–80% so that it can be used as the basic structural unit to construct a configurable passive optical angle power splitting device or wavelength multiplexer. The mechanism analysis involving the modulus and interference phase difference of multipole moments can provide a reference for the design and optimization of a phase-change antenna to realize a specific bidirectional scattering power splitter or wavelength multiplexer.

## Data Availability

Not applicable.

## Supplementary Materials

The following supporting information can be downloaded at https://www.mdpi.com/article/10.3390/nano12193305/s1, Figure S1: Electric (|E|) and magnetic (|H|) field distributions in the *xy*-plane of the V-shaped phase-change antenna at 3.6 μm wavelength; Figure S2: Multipole scattering cross sections and directivities of the V-shaped phase-change antenna at the crystallinity of 0% and 100%; Figure S3: Far-field radiation patterns of unit multipole moments; Figure S4: Interference far-field radiation patterns of the unit multipole moments with different phase differences; Figure S5: Directivities of interference far-field radiation according to the complex coefficients of major and minor multipole moments at 3.6 μm wavelength; Figure S6: D directivities of V-shaped phase-change antenna with different geometric angles.

## Abbreviations

The following abbreviations are used in this manuscript:

GST	Ge-Sb-Te
GSS4T1	Ge_2_Sb_2_Se_4_Te_1_
FEM	finite element method
PML	perfect matching layer
ED	electric dipole
MD	magnetic dipole
EQ	electric quadrupole
MQ	magnetic quadrupole
