# Supplementary material for: Reconfigurable Radiation Angle Continuous Deflection of All-Dielectric Phase-Change V-Shaped Antenna
Source: Nanomaterials (Basel). 2022 Sep 22;12(19):3305. doi: 10.3390/nano12193305 (PMC9565491; doi:10.3390/nano12193305)
Supplement: Supplementary file 1 [file nanomaterials-12-03305-s001.zip › nanomaterials-1873157-supplementary.pdf]

Supplementary Materials: Reconfigurable Radiation Angle Continuous Deflection of All-dielectric Phase-change V-shaped Antenna

Ping Tang [1], Qiao Tao [1], Shengde Liu [2], Jin Xiang [3], Liyun Zhong [1,*] and Yuwen Qin [1]

1. Electromagnetic Field Mode

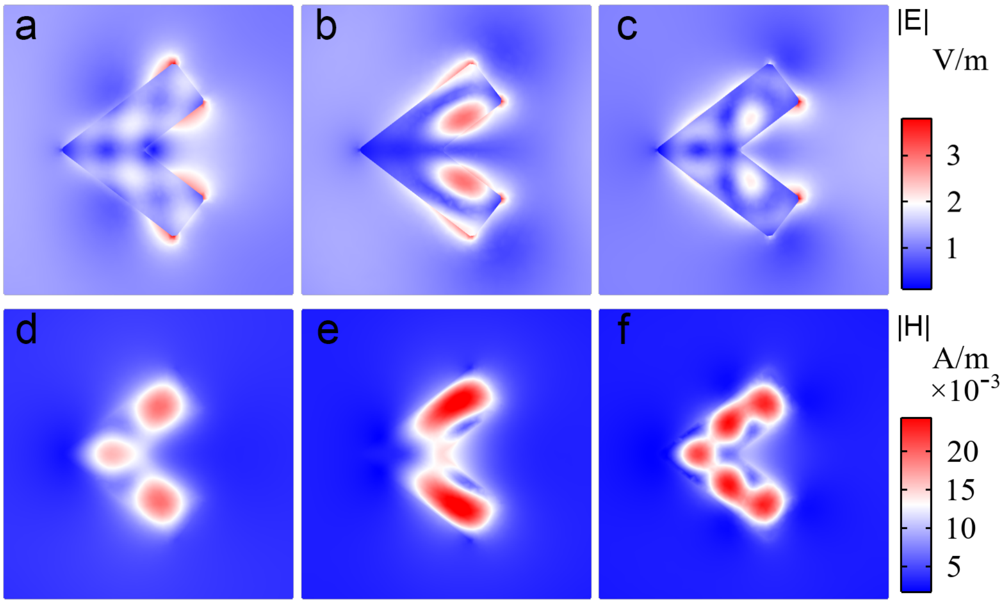

Figure S1. (**a–c**) Electric (|**E**|) and (**d–f**) magnetic (|**H**|) field distributions in the *xy*-plane of the V-shaped phase-change antenna at 3.6 μm wavelength with crystallinity of 20%, 50%, and 80%, respectively.

2. Amorphous and Crystalline V-shaped Phase-change Antennas

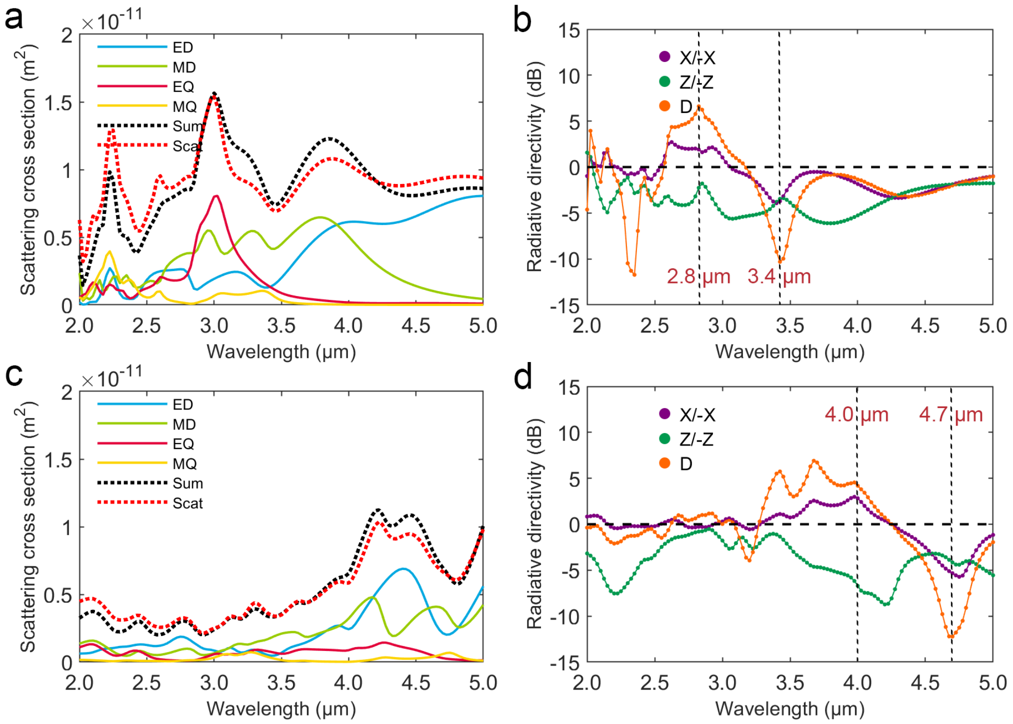

Figure S2. Multipole scattering cross sections and directivities of the V-shaped phase-change antenna in 2.0 to 5.0 μm wavelength range: (**a, c**) Scattering cross sections of ED, MD, EQ, MQ, their summation (Sum) and the total scattering cross sections calculated according to scattering filed (Scat) at the crystallinity of 0% and 100%, respectively; (**b, d**) Directivities of x-axis positive and negative (X/−X), z-axis positive and negative (Z/−Z), and the specific angle and window (D: θ_0=135°, δ=10°) at the crystallinity of 0% and 100%, respectively.

3. Far-field Radiation Patterns of Unit Multipole Scattering Moment

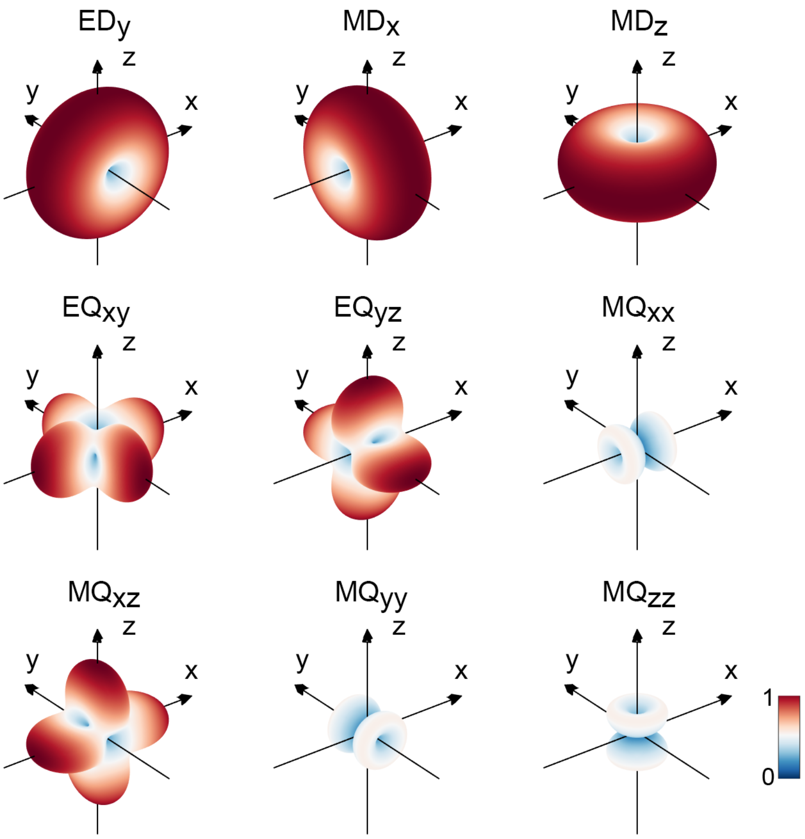

Figure S3. Far-field radiation patterns of unit ED_y, MD_x, MD_z, EQ_{xy}, EQ_{yz}, MQ_{xx}, MQ_{xz}, MQ_{yy}, and MQ_{zz}, respectively.

4. Interference Far-field Radiation of Unit Multipole Scattering Moments

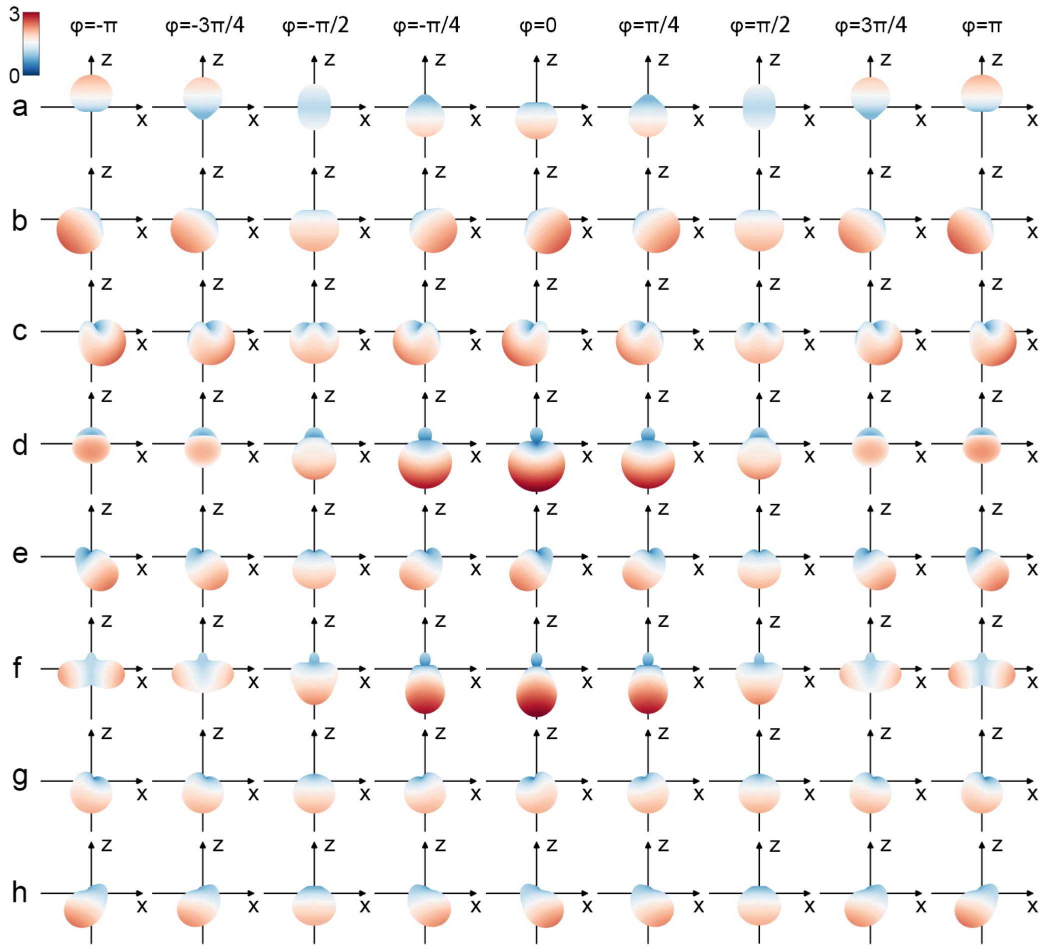

Figure S4. Interference far-field radiation patterns of the unit multipole moments with different phase differences: Far-field radiation patterns of (**a**) ED_y+exp$(i\varphi)MD_x$, (**b**) ED_y+MD_x+exp$(i\varphi)MD_z$, (**c**) ED_y+MD_x+exp$(i\varphi)EQ_{xy}$, (**d**) ED_y+MD_x+exp$(i\varphi)EQ_{yz}$, (**e**) ED_y+MD_x+exp$(i\varphi)MQ_{xx}$, (**f**) ED_y+MD_x+ exp$(i\varphi)MQ_{xz}$, (**g**) ED_y+MD_x+exp$(i\varphi)MQ_{yy}$, and (**h**) ED_y+MD_x+exp$(i\varphi)MQ_{zz}$.

The interference far-field radiation patterns of unit moments with different phase difference are shown in Figure S4. It can be seen that MD_x with different phase angle mainly affects the backward and forward scattering in the z-direction (Figure S4a). The calculated interference phase difference between MD_x and ED_y (see Figure 5b) varies between $-\pi/2$ and $\pi/2$ in crystallinity 0% to 100% range, so its main contribution is forward scattering to the $-z$ direction. In order to clarify the contribution of other unit multipole moments to the interference far-field, we analyzed the interference far-field radiation of ED_y+MD_x+exp$(i\varphi)MD_z$, ED_y+MD_x+exp$(i\varphi)EQ_{xy}$, ED_y+MD_x+exp$(i\varphi)EQ_{yz}$, ED_y+MD_x+exp$(i\varphi)MQ_{xx}$, ED_y+MD_x+exp$(i\varphi)MQ_{xz}$, ED_y+MD_x+exp$(i\varphi)MQ_{yy}$, and ED_y +MD_x+exp$(i\varphi)MQ_{zz}$, and the results are shown in Figure S4b–S4h, respectively. Distinctly, the MD_z, EQ_{xy}, MQ_{xx}, and MQ_{zz} deflect the interference far-field radiation direction; On the contrary, the contributions of EQ_{yz} and MQ_{xz} to the interference far-field radiation are symmetric about the yz-plane; And the intrinsic far-field radiation mode of unit MQ_{yy} (Figure S3) is none in xz-plane, so that its influence on D directivity can be ignored. Above results are consistent with the discussion based on Eq. 23 in the main text. Note that: 1) MQ_{xx} and MQ_{zz} belong to the third-order mode and the modulus of calculated scattering coefficient $\alpha_{MQ_{xx}}$ and $\alpha_{MQ_{zz}}$ are small relative to that of α_{MD_z} and $\alpha_{EQ_{xy}}$ (see Figure 4a), so as the contributions of MQ_{xx} and MQ_{zz} to the interference far field is relatively little; 2) It can be seen from Figure S4 that the intrinsic far field radiation patterns of unit MQ_{xx} and

MQ_{zz} are smaller than these of MD_z and EQ_{xy}, so MQ_{xx} and MQ_{zz} have a relatively minor impact. As a result, MD_z and EQ_{xy} make minor contributions to the radiation deflection while MD_z and EQ_{xy} are the key moments to the deflection of direction and make the major contributions to the D directivity. The detailed contributions of ED_y, MD_x, MD_z, and EQ_{xy} to the interference far-field radiation are discussed in the main text.

5. Directivity of Interference Far-field Radiation with Minor Contributions

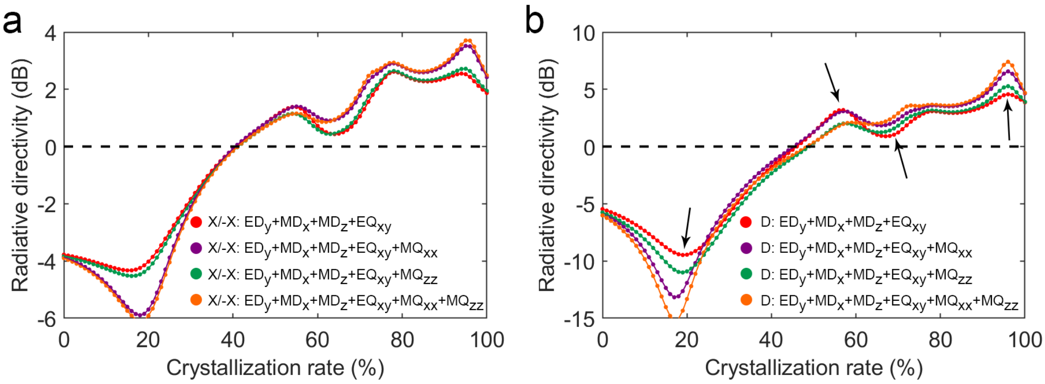

Figure S5. Directivities of interference far-field radiation according to the complex coefficients of major (ED_y, MD_x, MD_z, and EQ_{xy}) and minor (MQ_{xx} and MQ_{zz}) multipole moments at 3.6 μm wavelength: (**a**) X/−X directivity as a function of crystallinity; (**b**) D directivity as a function of crystallinity.

6. Effect of Geometric Angle on Directivity of V-shaped Phase-change Antenna

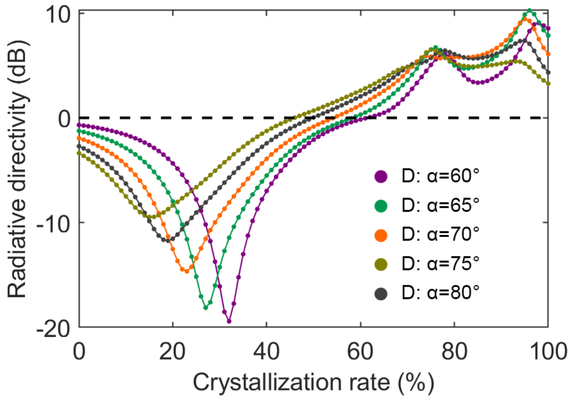

Figure S6. D directivities of V-shaped phase-change antenna with different geometric angles (α in Figure 1a), including α=60°, α=65°, α=70°, α=75°, and α=80°.
